# Supplementary material for: Genomic analysis identifies risk factors in restless legs syndrome
Source: medRxiv. 2023 Dec 20:2023.12.19.23300211. Preprint. [Version 1] doi: 10.1101/2023.12.19.23300211 (PMC10760278; doi:10.1101/2023.12.19.23300211)
Supplement: Supplement 4 [file NIHPP2023.12.19.23300211v1-supplement-4.pdf]

Supplementary information

Supplementary Table 1. Demographic characteristics of study samples in GWAS discovery cohort. Diagnostic questions[†] in surveys and the Personal Medical History* domain were used to identify cases.

Dataset	Cases (% females)	Controls (% females)	Mean age, cases/controls	Population	RLS identifier questions
All of Us WGS*	1,977 (74%)	10,137 (75%)	62 (± 14), 62 (± 15)	United States	Has a doctor or health care provider ever told you that you have or had any of the following brain and nervous system conditions? (Restless legs syndrome)
All of Us GDA*	1,973 (73%)	11,523 (70%)	62 (± 14), 62 (± 15)		
CARTaGENE[†]	921 (67%)	13,07 (60%)	56 (± 8), 56 (± 8)	Quebec (Canada)	Do you have restless legs syndrome?<br>Generally, your discomforts are worse…at rest/during activity/no difference/prefer not to answer/do not know<br>Generally, your discomforts are relieved by… walking or movement/immobility or relaxation/prefer not to answer/do not know<br>Generally, your discomforts are worse… in the morning/in the afternoon/evening, bedtime, night/no difference/prefer not to answer/do not know
Canadian Longitudinal Study on Aging[†]	4,980 (60%)	15,990 (47%)	63 (± 10), 63 (± 10)	Canada	Do you have, or have you sometimes experienced, a recurrent need or urge to move your legs while sitting or lying down?
Total number	9,851	38,957			

medRxiv preprint doi: https://doi.org/10.1101/2023.12.19.23300211; this version posted December 20, 2023. The copyright holder for this preprint (which was not certified by peer review) is the author/funder, who has granted medRxiv a license to display the preprint in perpetuity.
This article is a US Government work. It is not subject to copyright under 17 USC 105 and is also made available for use under a CC0 license.

Supplementary Table 2. Gene mapping in the RLS risk regions. Annotation was performed in FUMA (v.1.5.1). It is provided as an Excel file.

Supplementary Table 3. Adjusted covariates in the logistic regression test. Covariates were selected using the MASS stepwise function. PC, principal component.

Dataset	List of the covariates
CARTaGENE	sex, age, PC1, PC2, PC4, PC7, PC10
Canadian Longitudinal Study on Aging	sex, age, PC2, PC6
All of Us	sex, age, PC3, PC8, PC9, PC10
All of Us WGS	sex, age, PC2, PC3, PC7, PC10

Supplementary Table 4. Replication of other known RLS risk variants in our meta-analysis.

Chr	Position (hg38)	EA/OA	Closest gene(s)	Direction	OR	*P*
2	3,986,856	G/A	*DCDC2C*	++++	1.08 (1.05–1.12)	3.03×10^{-6}
2	158,343,323	T/C	*CCDC148*	----	0.93 (0.90–0.97)	3.14×10^{-4}
2	189,584,800	T/A	*SLC40A1*	----	0.96 (0.93–0.99)	0.016
2	67,842,758	A/C	*C1D*	NA	NA	NA
3	3,406,460	T/A	*CRBN*	----	0.94 (0.90–0.97)	3.69×10^{-4}
3	130,816,723	G/A	*ATP2C1*	++++	1.07 (1.04–1.11)	6.25×10^{-5}
5	171,001,975	T/C	*RANBP17*	NA	NA	NA
6	37,522,755	G/A	*CCDC167*	++++	1.09 (1.05–1.14)	1.75×10^{-5}
9	9,290,311	T/C	*PTPRD, PTPRD-AS1*	----	0.95 (0.91–0.98)	1.76×10^{-3}
11	8,313,948	A/G	*LMO1*	NA	NA	NA
13	72,274,018	T/G	*DACH1*	++++	1.07 (1.03–1.11)	6.40×10^{-4}
15	47,068,169	T/G	*SEMA6D*	----	0.90 (0.85–0.95)	2.01×10^{-4}
15	35,916,797	T/A	*DPH6*	----	0.90 (0.84–0.96)	3.01×10^{-3}
17	48,695,414	A/G	*PRAC1*	++++	1.04 (1.00–1.08)	0.08
18	44,290,278	T/C	*SETBP1*	++++	1.05 (1.01–1.09)	0.01
18	59,943,413	T/C	*PMAIP1*	NA	NA	NA

medRxiv preprint doi: https://doi.org/10.1101/2023.12.19.23300211; this version posted December 20, 2023. The copyright holder for this preprint (which was not certified by peer review) is the author/funder, who has granted medRxiv a license to display the preprint in perpetuity.
This article is a US Government work. It is not subject to copyright under 17 USC 105 and is also made available for use under a CC0 license.

20	64,164,052	G/A	*MYT1*	----	0.91 (0.88–0.94)	1.17×10^{-7}

Supplementary Table 5. Causal posterior probabilities for genes in 90%-credible sets for restless legs syndrome transcriptome-wide association study signals. PIP, posterior inclusion probability.

**Gene**	**Region**	**Number of genes in the region**	**Proxy tissue**	**Method**	**PIP**
PRMT6	1:105545220-107867043	16	spinal cord	lasso	0.06
STEAP2	7:88195689-91032469	35	spinal cord	susie	0.06
GTPBP10	7:88195689-91032469	35	nucleus accumbens	lasso	0.02
PTPRD-AS1	9:8456299-9166403	7	spinal cord	susie	0.13
SKOR1	15:66802429-68725660	30	nucleus accumbens	susie	0.69

Supplementary Table 6. Regulome-wide association study (RWAS) results. RWAS was done using MAGMA (v.1.06). Bonferroni correction threshold for each tissue was defined based on the number of enhancers tested. It is provided as an Excel file.

Supplementary Table 7. Gene sets and pathways identified by EnrichR using GO biological process and Reactome pathway datasets.

medRxiv preprint doi: https://doi.org/10.1101/2023.12.19.23300211; this version posted December 20, 2023. The copyright holder for this preprint (which was not certified by peer review) is the author/funder, who has granted medRxiv a license to display the preprint in perpetuity.
This article is a US Government work. It is not subject to copyright under 17 USC 105 and is also made available for use under a CC0 license.

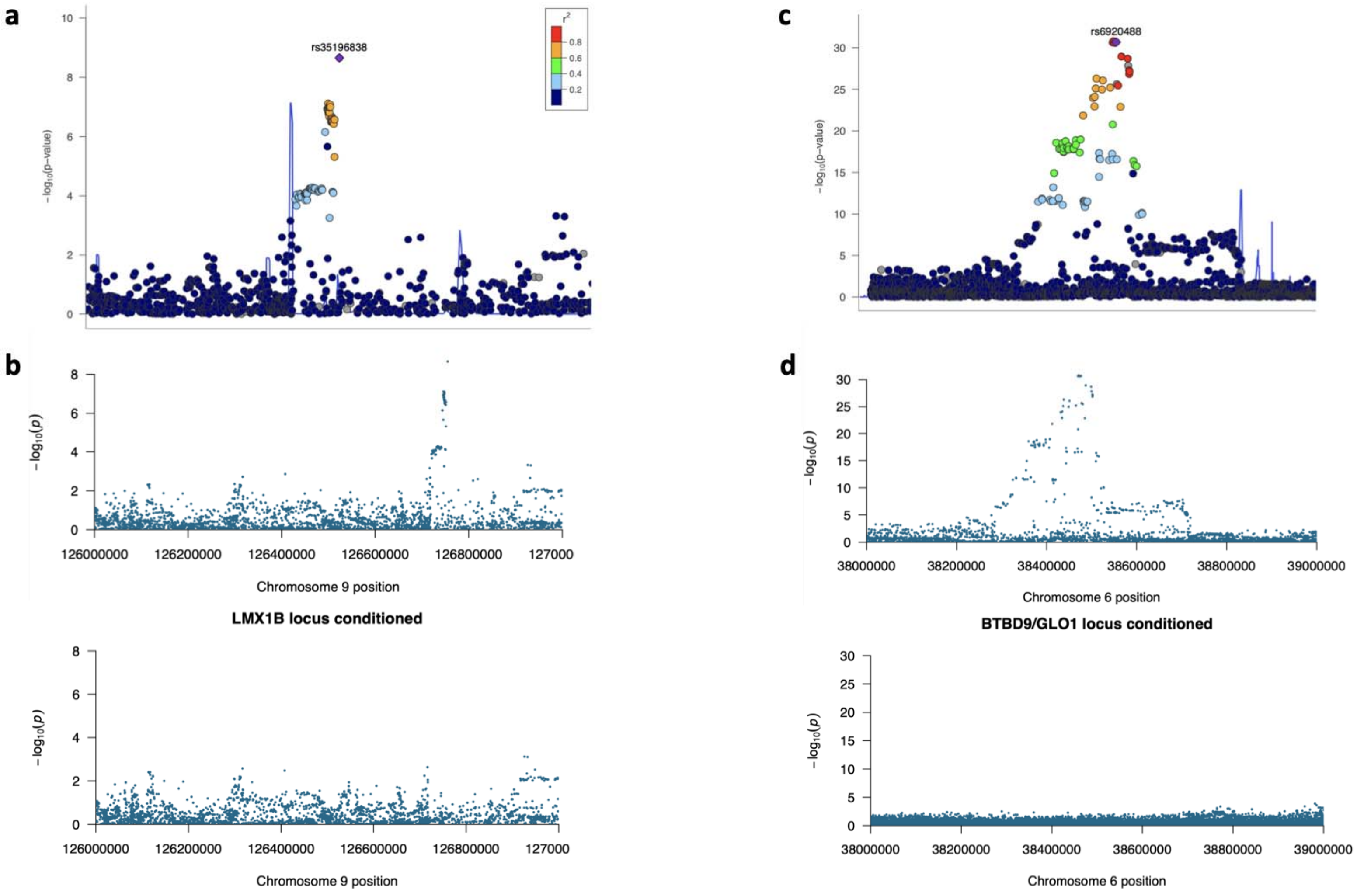

Supplementary Figure 1. a. LocusZoom plot for chr9:126755162:C:A, **b.** Conditional analysis at the *LMX1B* locus (before and after conditioning), **c.** LocusZoom plot for chr6:38476264:A:G, **d.** Conditional analysis at the *BTBD9/GLO1* locus (before and after conditioning).

medRxiv preprint doi: https://doi.org/10.1101/2023.12.19.23300211; this version posted December 20, 2023. The copyright holder for this preprint (which was not certified by peer review) is the author/funder, who has granted medRxiv a license to display the preprint in perpetuity. This article is a US Government work. It is not subject to copyright under 17 USC 105 and is also made available for use under a CC0 license.

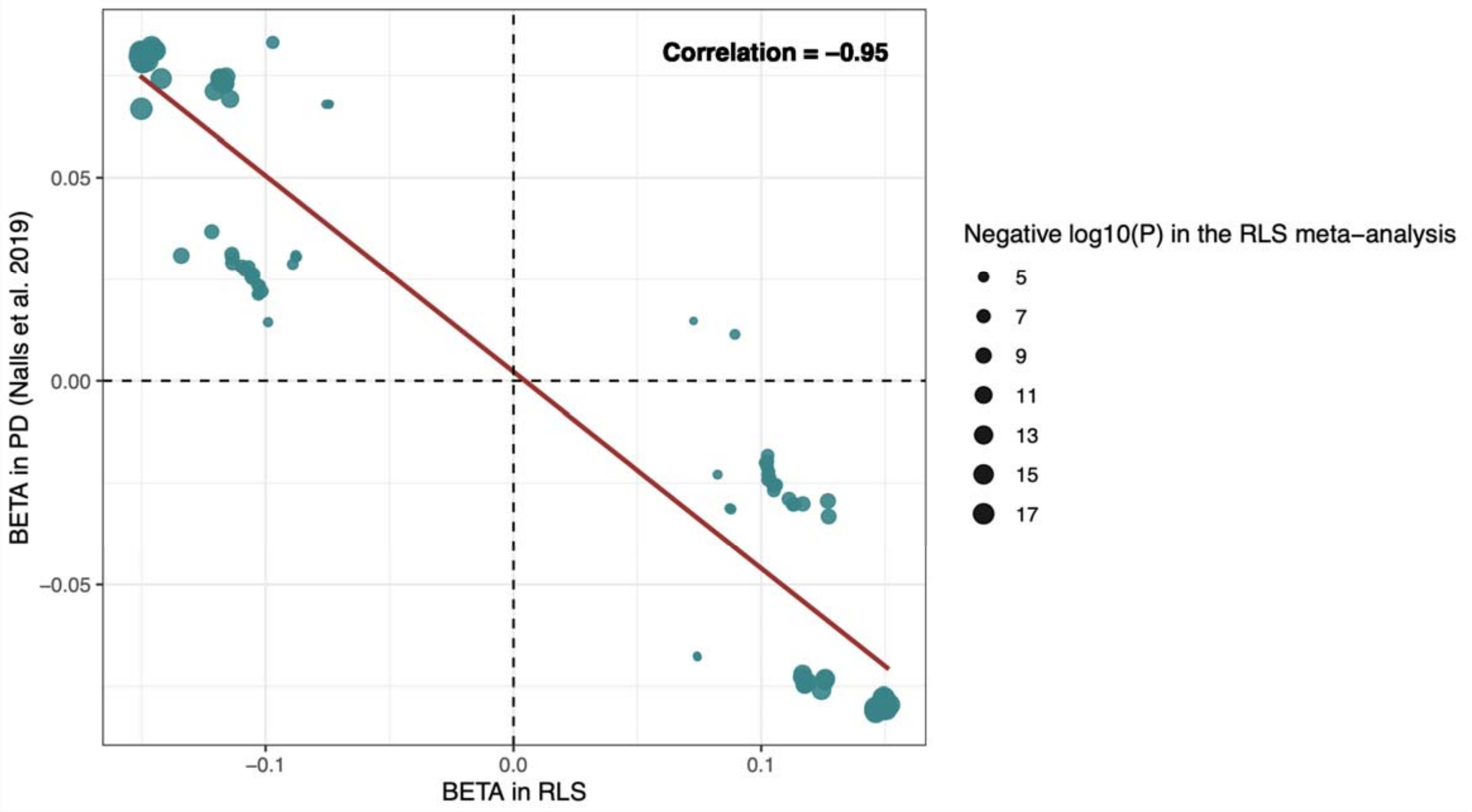

Supplementary Figure 2. Beta-beta plot for *TOX3* variants in the RLS meta-analysis and Parkinson's disease meta-analysis. We compared the effect of the *TOX3* variants ($P < 1 \times 10^{-4}$) identified in our discovery meta-analysis with the recent Parkinson's disease GWAS by Nalls et al. [ref]. For the top significant variant in Parkinson's disease (16:52602330:C:A, hg38), we included the beta value from the meta-analysis, whereas, for the rest of the variants, we included summary statistics without 23andMe datasets. A negative correlation was found between the effects of *TOX3* variants in RLS and PD (The correlation coefficient is -0.95, and the Pearson correlation R^2 is 91).

medRxiv preprint doi: https://doi.org/10.1101/2023.12.19.23300211; this version posted December 20, 2023. The copyright holder for this preprint (which was not certified by peer review) is the author/funder, who has granted medRxiv a license to display the preprint in perpetuity. This article is a US Government work. It is not subject to copyright under 17 USC 105 and is also made available for use under a CC0 license.
